# Study on novel modified large mesoporous silica FDU-12/polymer matrix nanocomposites for adsorption of Pb(II)

**DOI:** 10.1371/journal.pone.0245583

**Published:** 2021-01-22

**Authors:** Hamed Ghaforinejad, Hossein Mazaheri, Ali Hassani Joshaghani, Azam Marjani

**Affiliations:** 1 Department of Chemical Engineering, Arak Branch, Islamic Azad University, Arak, Iran; 2 Department for Management of Science and Technology Development, Ton Duc Thang University, Ho Chi Minh City, Viet Nam; 3 Faculty of Applied Sciences, Ton Duc Thang University, Ho Chi Minh City, Viet Nam; University of Southern Denmark, DENMARK

## Abstract

In this study, porous methacrylate-modified FDU-12/poly(methyl methacrylate) and amine-modified FDU-12/Nylon 6 nanocomposites were synthesized *via* a facile solution casting protocol. The physicochemical properties of the prepared materials were studied using various characterization techniques including Fourier transform-infrared spectroscopy, field emission-scanning electron microscopy, transmission electron microscopy, and nitrogen adsorption/desorption. After characterization of the materials, the prepared nanocomposites were applied as novel adsorbents for the removal of Pb(II) from aqueous media. In this regard, the effect of various parameters including solution pH, adsorbent amount, contact time, and initial concentration of Pb(II) on the adsorption process was investigated. To study the mechanism of adsorption, kinetic studies were conducted. The kinetic models of pseudo-first-order, pseudo-second-order, Elovich, and intraparticle diffusion were employed. The results revealed that the adsorption of Pb(II) onto methacrylate-modified FDU-12/poly(methyl methacrylate) and amine-modified FDU-12/Nylon 6 adsorbents followed the pseudo-second-order kinetic model. Also, different isotherms including Langmuir, Freundlich, and Dubinin-Radushkevich were applied to evaluate the equilibrium adsorption data. Langmuir isotherm provided the best fit with the equilibrium data of both adsorbents with maximum adsorption capacities of 99.0 and 94.3 mg g^-1^ for methacrylate-modified FDU-12/poly(methyl methacrylate) and amine-modified FDU-12/Nylon 6, respectively, for the removal of Pb(II).

## 1. Introduction

Today, the discharge of environmental contaminants especially heavy metals (Pb(II), Cd(II), Hg(II), Cr(VI), etc.) in the environment caused drastic concerns about the health of individuals exposed to them. Heavy metals are an important class of environmental contaminants due to their high toxicity, carcinogenicity, nonbiodegradability, and bioaccumulative nature [1]. As an extremely toxic member of heavy metals, Pb(II) has bad effects on the kidney, liver, reproductive system, brain functions, and basic cellular processes. It can cause mental retardation, insomnia, irritability, seizures, and renal damages. The major sources of Pb(II) in the environment are ore and metals processing, production of fertilizers, pigments, batteries, and leaded aviation fuels. Every day, health-related organizations and environmentalists express their deep concern about these contaminants. Because of these concerns, especially about human health, the US Environmental Protection Agency has announced a limit on the amount of heavy metals in soil and water. Thus, contaminant uptake, especially from aquatic media is urgent. Widespread researches have been conducted to introduce and develop decontamination methods. They are included chemical precipitation, electrochemical remediation, membrane filtration, ion exchange, solvent extraction, coagulation, adsorption, etc. Among them, the adsorption strategy is popular for effective water treatment owing to its several advantages. This technique provided a simple, relatively fast, and low-cost methodology with high efficiency and flexibility [2]. The method is also eco-friendly in large-scale manufacturing and can combine with other conventional remediation methods.

Considering intensive developments in nanotechnology especially in the fields of catalysis [3–9], extraction [10–14], carrier materials [15], and adsorption [16–21], polymer matrix nanocomposites have attracted a great deal of attention in the field of adsorption. The use of nanosized particles in the polymer matrixes provides high-performance hybrid nanocomposite materials with widespread applications. These materials combine ductility and flexibility of organic polymer matrix with the benefits of inorganic nanofiller such as high thermal and mechanical stability, and high surface area. On the other hand, the abundant functional groups (hydroxyl, carbonyl, phenyl, amine, etc.) on the surface of polymer matrix nanocomposites results in adsorbents with a good affinity toward adsorption of heavy metals [1, 22, 23]. Recently, mesoporous silica materials have attracted much attention to use as adsorbent or a part of adsorbent in polymer matrix nanocomposites due to their unique properties. They provide high surface area, functionalizable surface, and tailorable pore dimensions which make them a good candidate for adsorption processes. Over the past years, a plethora of mesoporous silica materials (examples include MCMs, SBAs, KCC-1, KITs, FDUs) with a wide range of pore geometries and particle morphologies have been introduced and used for widespread applications, especially for adsorption [24, 25]. Generally, they can be classified as 2D (e.g. SBA-15, MCM-41) and 3D (e.g. MCM-48, SBA-16, KIT-6, FDU-12) architecture based on the pore symmetry [26, 27].

As a three-dimensional large mesoporous silica, FDU-12 possesses a highly ordered structure with a superior 3D channel which makes it ideal for mass transfer and diffusion of guest molecules. It has unique properties of well-ordered pore structure, high specific surface area, ultra-large pore diameter (10–26 nm), and adjustable pore size [28, 29]. FDU-12 can be synthesized with different pore size distributions as reported in recent studies [30, 31]. To expand the application of mesoporous silica materials (e.g. FDU-12), changing hydrophobicity, and tailoring surface characteristics, the incorporation of organic functional groups onto the ordered structure of the material with uniform distribution is a widely used choice. In the case of nanocomposites, functionalization of the inorganic filler with a suitable functional group could help linking up the filler with the polymer matrix through functional groups and also more effective dispersion and penetration of the polymer chains in the porous structure of the filler. Since the surface of mesoporous silica materials is densely populated with hydroxyl groups, surface functionalization with a variety of organic moieties seems to be relatively easy [32]. In this context, several studies reported surface functionalization of mesoporous silica materials such as SBA-15, MCM-41, and KCC-1 with various organic groups [1, 2, 21, 23, 33, 34]. In comparison, there are few reports regarding the functionalization of mesoporous silica FDU-12 [35–39].

In the case of polymer matrix nanocomposites, various organic polymers have been applied for the preparation of polymer-based nanocomposites containing mesoporous materials. As a low density, transparent, thermoplastic, and rigid polymer, poly(methyl methacrylate) (PMMA) provided good flexibility and low cost. This versatile amorphous polymer has also excellent electrical and mechanical properties, and good resistance to non-polar solvents, acidic and alkaline solutions, and inorganic reagents [40, 41]. Another versatile polymer used in polymer matrix nanocomposites is Nylon. Nylon polymer is an electron-rich and polar synthetic polyamide composed of microfibrils that are interconnected forming a three-dimensional network with a porous structure [22, 42]. Among various types of commercial Nylons, Nylon 6 and Nylon 6,6 continue to be the most popular types. Nylon 6 fibers have high tensile strength and provided highly abrasion resistance. This polymer is also resistant to various types of chemicals such as acids and alkalis.

Herein, we present the synthesis and characterization of two novel modified mesoporous silica FDU-12 materials using silane coupling agents of 3-(triethoxysilyl)propyl methacrylate and *N*^1^-(3-trimethoxysilylpropyl)diethylenetriamine. The prepared materials were then used as nanofiller for the preparation of poly(methyl methacrylate)- and Nylon 6-based nanocomposites. The nanocomposites were synthesized *via* a facile and fast method and characterized by various characterization techniques. To study the applicability of the prepared nanocomposites for adsorption purposes, the nanocomposites were used as novel adsorbents for removal of Pb(II) ions from aqueous solutions. Kinetic studies were also conducted for the two prepared materials. Our results showed the potential of methacrylate- and amine-functionalized FDU-12 for the adsorption of Pb(II) ions as a model of heavy metals from aqueous media.

## 2. Experimental

### 2.1. Materials and reagents

In this study, toluene (99%), absolute ethanol (99.5%), formic acid (99%), hydrochloric acid (37%), acetic acid (99.5%), *ortho*-phosphoric acid (85%), tetraethyl orthosilicate (TEOS), boric acid (99.5%), potassium chloride (99.5%), sodium hydroxide (97%), and lead(II) nitrate (99.5%) were obtained from Merck (Darmstadt, Germany). Poly(methyl methacrylate) (PMMA, average M_w_ ~120,000), Nylon 6 (NY6), Pluronic F-127 (Mw ~ 12600 Da), 1,3,5-trimethylbenzene (TMB, 98%), and *N*^1^-(3-trimethoxysilylpropyl)diethylenetriamine were purchased from Sigma-Aldrich (St. Louis, MO, USA). 3-(triethoxysilyl)propyl methacrylate (98%) was obtained from TCI (Europe). Deionized water was prepared using a water purification system (Oklahoma, USA). The stock standard solution of Pb(II) (1000 mg L^−1^) was prepared in water. Working standard solutions were prepared by diluting the stock solution. The Brighton-Robinson buffer system was used for the preparation of aqueous solutions with different pHs.

### 2.2. Instruments

The Fourier transform-infrared (FT-IR) spectra were recorded using a Thermo Nicolet Avatar 330 FT-IR spectrometer (USA) between 4000 and 400 cm^-1^ with a resolution of 4 cm^-1^. The surface morphology of the samples was examined using a MIRA3 TESCAN-XMU field emission-scanning electron microscope (FE-SEM, Czech Republic). Transmission electron microscopy (TEM) analysis was performed on a Philips CM 120 microscope (Philips Electronics, Eindhoven, The Netherlands). For the analysis, the samples were dispersed in 2-propanol and a drop of the suspension was put on a carbon-coated nickel grid. Nitrogen adsorption/desorption analysis was performed on a Belsorp-mini II (BEL Japan Inc., Osaka, Japan) at 77 K. A flame atomic absorption spectroscopy (FAAS, Agilent, Model 240FS AA, USA) was used for Pb quantification in the samples. In the case of samples with a low level of Pb, an inductively coupled plasma-optical emission spectrometer (ICP-OES, PerkinElmer, Optima 7300 DV, USA) was applied. A 100 W ultrasonic liquid processor (MISONIX XL-2000 Series, Raleigh, North Carolina, USA) was used for sonochemical reactions.

### 2.3. Synthesis and surface modification of large mesoporous silica FDU-12

The large mesoporous silica FDU-12 was synthesized according to the previous reports [29, 43] with some modifications. Briefly, 3.0 g of Pluronic F-127 was dissolved in 120 mL of 2.0 mol L^-1^ hydrochloric acid. After 15 min, 7.5 g of potassium chloride was added to the solution. Then, 3.0 g of TMB was added and the mixture was stirred at 288 K for 2 h. Afterward, 12.5 g of TEOS was added to the solution and the mixture was heated to 383 K in an autoclave and remained at this temperature for 72 h. The white solid product was collected and washed with water and ethanol and oven-dried at 343 K for 24 h. The calcination of the material was performed at 823 K for 5 h.

Two silane coupling agents including 3-(triethoxysilyl)propyl methacrylate and *N*^1^-(3-trimethoxysilylpropyl)diethylenetriamine were used to surface modification of the prepared FDU-12 through the post-synthesis modification technique. Typically, 0.5 g of the synthesized FDU-12 and 0.5 mL of silane coupling agent were added to 40 mL of dried toluene. The mixture was refluxed at 383 K for 24 h. The resultant product was then isolated through a Büchner funnel and washed with toluene and ethanol, and oven-dried at 343 K for 24 h. The FDU-12 samples modified with 3-(triethoxysilyl)propyl methacrylate and *N*^1^-(3-trimethoxysilylpropyl)diethylenetriamine were denoted as FDU-12-MA and FDU-12-TA, respectively.

### 2.4. Preparation of FDU-12-MA/PMMA and FDU-12-TA/NY6

For the preparation of FDU-12-MA/PMMA nanocomposite with FDU-12-MA content of 3.0 wt%, 4.85 g of PMMA was dissolved in 40 mL of dry toluene (with the help of heating at 80°C under nitrogen atmosphere) and the mixture was sonicated for 15 min. Afterward, 0.15 g (3.0 wt%) of the prepared FDU-12-MA was added to 10 mL of dry toluene and the mixture was sonicated for 15 min. Then, the FDU-12-MA mixture was added to the polymer solution with mechanical stirring, sonicated for 15 min, and refluxed for 24 h. After cooling down to room temperature, the resultant mixture was sonicated for another 15 min, poured into a clean glass Petri dish, and dried at room temperature for 5 h. The FDU-12-MA/PMMA nanocomposite was washed with pure water, dried, and used for adsorption experiments.

In the case of FDU-12-TA/NY6, 4.85 g of NY6 was dissolved in 40 mL of formic acid (with the help of heating at 80°C under nitrogen atmosphere) and the mixture was refluxed under nitrogen atmosphere for 6 h to obtain a clear solution. Afterward, 0.15 g (3.0 wt%) of the prepared FDU-12-TA was added to 10 mL of formic acid and the mixture was sonicated for 15 min. Then, the FDU-12-TA mixture was added dropwise to the NY6 solution, sonicated for 15 min, and refluxed for 12 h. After refluxing, the mixture was sonicated for another 15 min, cast to a clean glass Petri dish, and dried at room temperature. The FDU-12-TA/NY6 nanocomposite was washed with pure water, dried, and used for adsorption experiments.

### 2.5. Adsorption experiments

Batch adsorption experiments were carried out to study the adsorption behavior of Pb(II) ions onto the FDU-12-MA/PMMA and FDU-12-TA/NY6. For each experiment, 10 mL of an aqueous standard solution of Pb(II) with the desired concentration was exposed to the accurately weighed amount of adsorbent in 20 mL polyethylene containers. The adsorption process was performed at 180 rpm and 298 K for 24 h. After that, the adsorbent was separated from the solution and the concentration of Pb(II) in the solution was quantified by the means of FAAS or ICP-OES. The removal efficiency (*RE*) was computed using the following equation (Eq 1):

$$RE\;(\%)=\frac{C_{i}-C_{e}}{C_{i}}\times 100$$ (1)

where *C*_*i*_ and *C*_*e*_ are the initial and equilibrium concentration of the metal ion in the solution (mg L^-1^), respectively. The adsorption capacity (*q*_*e*_, mg g^-1^) was calculated according to the following equation (Eq 2):

$$q_{e}=\left(\frac{C_{i}-C_{e}}{W}\right)\times V$$ (2)

Where *V* is the solution volume (mL) and *W* refers to the adsorbent amount (mg).

## 3. Results and discussion

### 3.1. Synthesis and characterization

Considering the relatively inert and hydrophobic nature of the pristine FDU-12, and also the tendency of nanoparticles to agglomerate, the surface functionalization is necessary to provide a homogenous dispersion of prepared mesoporous silica in the polymer matrix for the preparation of polymer nanocomposites. In the present study, the surface of the prepared pristine FDU-12 was treated with two different silane coupling agents of 3-(triethoxysilyl)propyl methacrylate and *N*^1^-(3-trimethoxysilylpropyl)diethylenetriamine. These coupling agents improve the interfacial interaction of inorganic filler and organic polymer matrix. The methacrylate- and amine-functionalized FDU-12 materials were then applied as nanofiller for the preparation of two nanocomposites using PMMA and NY6 as organic polymers.

**Fig 1:**
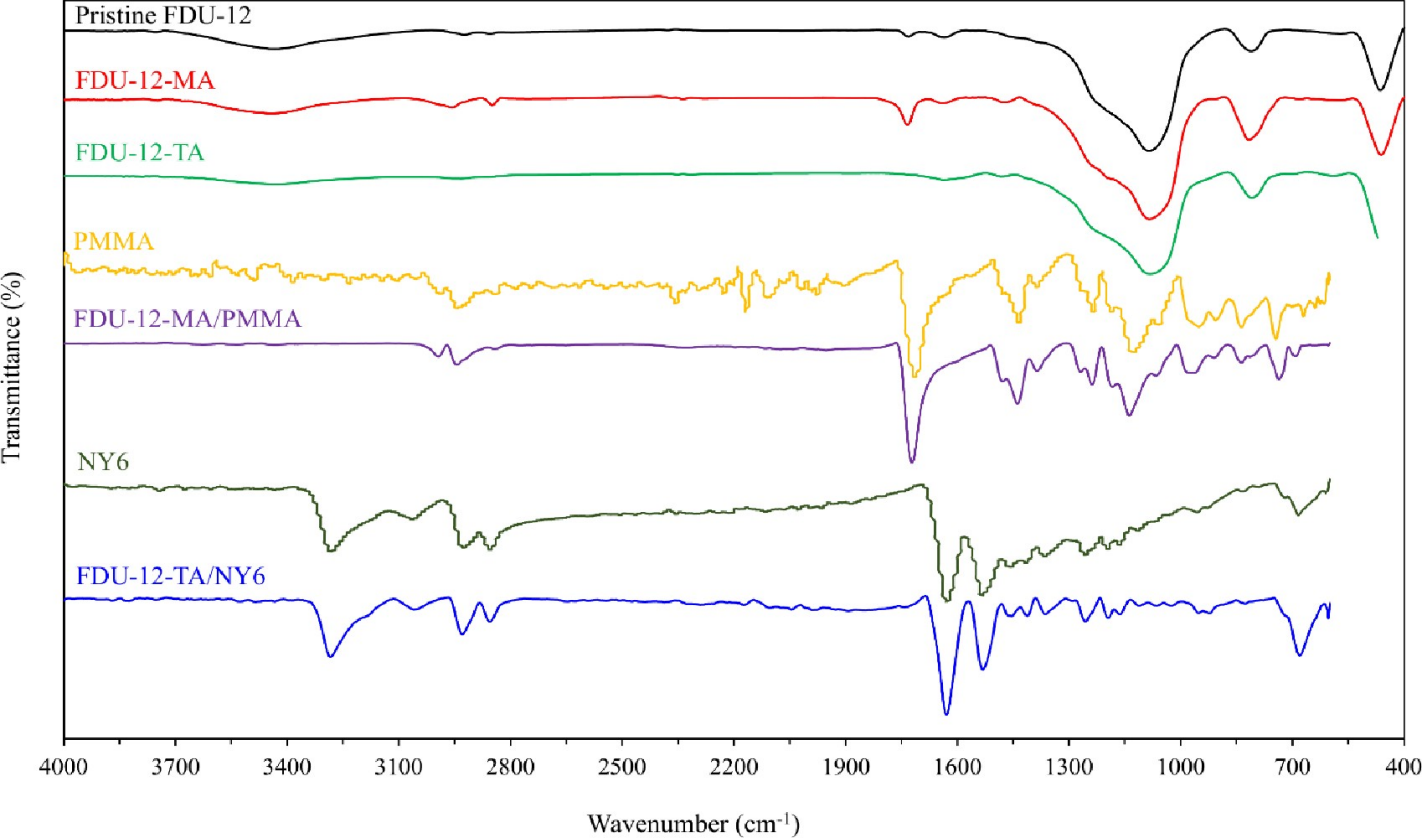
FT-IR spectra of pristine FDU-12, FDU-12-MA, FDU-12-TA, pristine PMMA, FDU-12-MA/PMMA, pristine NY6, and FDU-12-TA/NY6.

The chemical structure and morphology of the prepared materials were examined by FT-IR, FE-SEM, TEM, and N_2_ adsorption/desorption techniques. The FT-IR spectra of pristine FDU-12, FDU-12-MA, FDU-12-TA, pristine PMMA, FDU-12-MA/PMMA, pristine NY6, and FDU-12-TA/NY6 are shown in Fig 1. In the case of pristine FDU-12, the bands at 463 and 810 cm^−1^ are attributed to the bending and symmetric stretching vibration of Si–O–Si. Also, the band at 1078 cm^−1^ corresponds to the asymmetric stretching vibration of the Si–O–Si bond [28, 43]. The broadband around 3435 cm^−1^ corresponds to O–H stretching of the surface silanol groups and the physically adsorbed water molecules. The band centered at 1633 cm^−1^ is attributed to the bending vibration of H–O–H. For FDU-12-MA and FDU-12-TA, in addition to the bands observed for pure FDU-12, new bands related to the organic part of the silane coupling agent are appeared. The bands located at 2957 and 2849 cm^−1^ are assigned to the C–H stretching vibrations of CH_2_ and the band at 1471 cm^−1^ is corresponding to the bending vibration of the C–H. In the case of FDU-12-MA, the new band at 1733 cm^−1^ is characteristic of the C = O bond of the silane coupling agent. For FDU-12-TA, the broadband at 3200–3500 cm^−1^ indicated the presence of–OH and–NH_2_ groups on the surface of FDU-12-TA. These data indicated successful modification of pure FDU-12 with 3-(triethoxysilyl)propyl methacrylate and *N*^1^-(3-trimethoxysilylpropyl)diethylenetriamine. The FT-IR spectrum of FDU-12-MA/PMMA composite shows absorption bands at 2994 and 2944 cm^−1^ which correspond to C–H asymmetric stretching in CH_3_ and CH_2_ groups, respectively. Also, the peak located at 2842 cm^−1^ is related to the C–H symmetric stretching in CH_3_. The characteristic band at 1722 cm^−1^ which corresponds to the C = O bond is also observed. These bands in addition to the new bands observed in the spectrum of FDU-12-MA/PMMA correspond to different modes of CH_2_ and CH_3_ vibrational modes indicated the presence of PMMA in the structure of the prepared nanocomposite which is in accordance with other reports [23, 44]. Also, the similarities between the spectra of pristine PMMA and FDU-12-MA/PMMA are quite obvious. In the case of the C = O bond, the stretching vibration was observed at 1715 and 1722 cm^−1^ for pristine PMMA and FDU-12-MA/PMMA, respectively. The shifts of C = O stretching vibrations may be due to the interaction of modified nanofiller with polymer matrix (hydrogen bonding interactions) which decreases the strength of the C = O bond in the polymer [45–47]. For FDU-12-TA/NY6, the characteristic peaks at 3284, 2856 & 2929 cm^−1^ correspond to the stretching vibration of N-H bond and C-H stretching vibrations of the NY6. Also, the peaks located at 1631 and 1532 cm^−1^ are attributed to the stretching vibration of carbonyl groups and N-H bending vibration which all are related to NY6 [1]. The similarities between the spectra of pristine NY6 and FDU-12-TA/NY6 are observable. In the case of C = O bond, the stretching vibration observed at 1628 and 1631 cm^−1^ for pristine NY6 and FDU-12-TA/NY6, respectively. This shift also may be due to the hydrogen bonding interactions between nanofiller and polymer matrix which decreases the strength of the C = O bond in the polymer.

**Fig 2:**
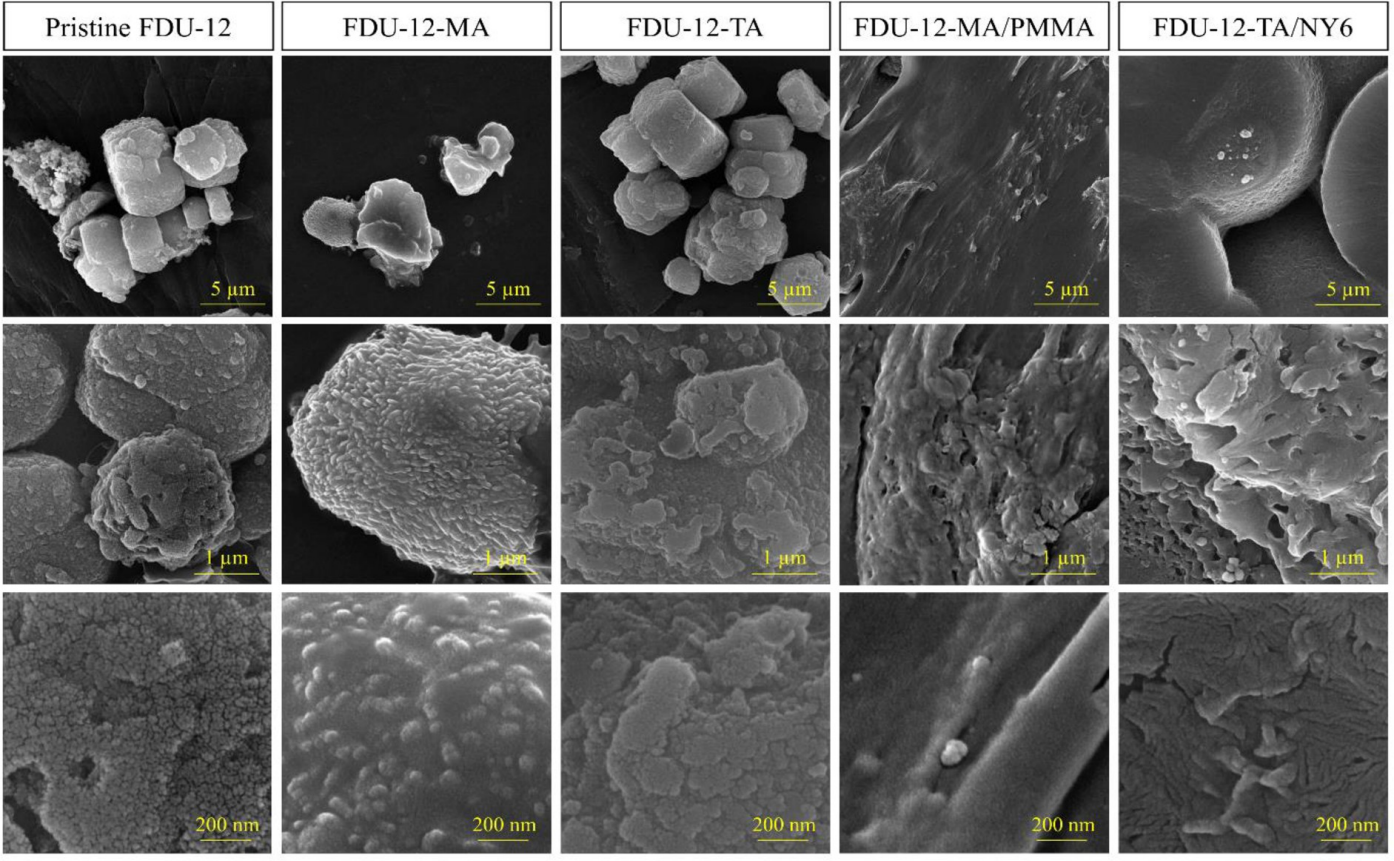
FE-SEM images of pristine FDU-12, FDU-12-MA, FDU-12-TA, FDU-12-MA/PMMA, and FDU-12-TA/NY6 at three magnifications.

**Fig 3:**
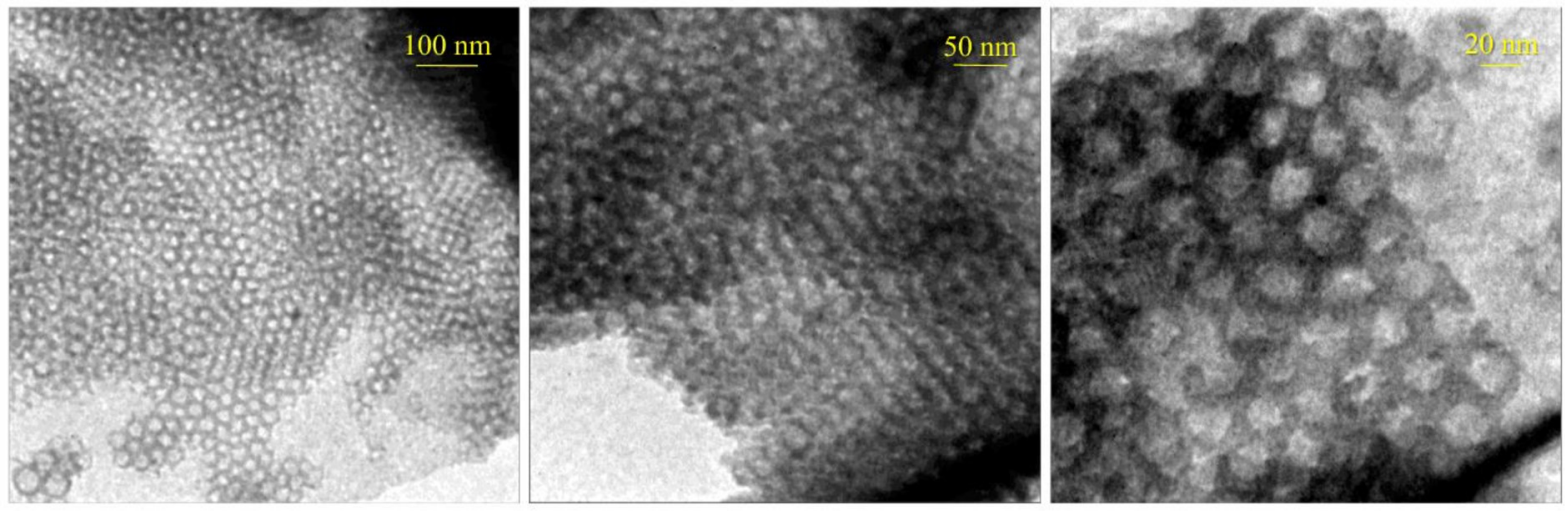
TEM images of pristine FDU-12.

The morphology of the prepared materials was studied *via* FE-SEM and TEM techniques. The FE-SEM images of pristine FDU-12, FDU-12-MA, FDU-12-TA, FDU-12-MA/PMMA, and FDU-12-TA/NY6 are shown in Fig 2. The FE-SEM image of the pristine FDU-12 and modified samples (FDU-12-MA, FDU-12-TA) showed a relatively regular hexagonal prism morphology which is a typical characteristic morphology of FDU-12 [43, 48]. This indicates that the morphology of the FDU-12 was retained after modification with the used silane coupling agents. At higher magnifications, the growing crystals are visible. In the case of polymer composites, the modified FDU-12 particles are visible on the surface of nanocomposites suggesting that the mesoporous material is incorporated in the polymer matrix. The TEM images of pristine FDU-12 are shown in Fig 3. A well-ordered structure is seen. As reported in the literature [48–50], the FDU-12 exhibited ordered mesopore arrangement characteristics. In the images, the large cage units with regular alignment in the highly ordered lattice are obvious.

**Fig 4:**
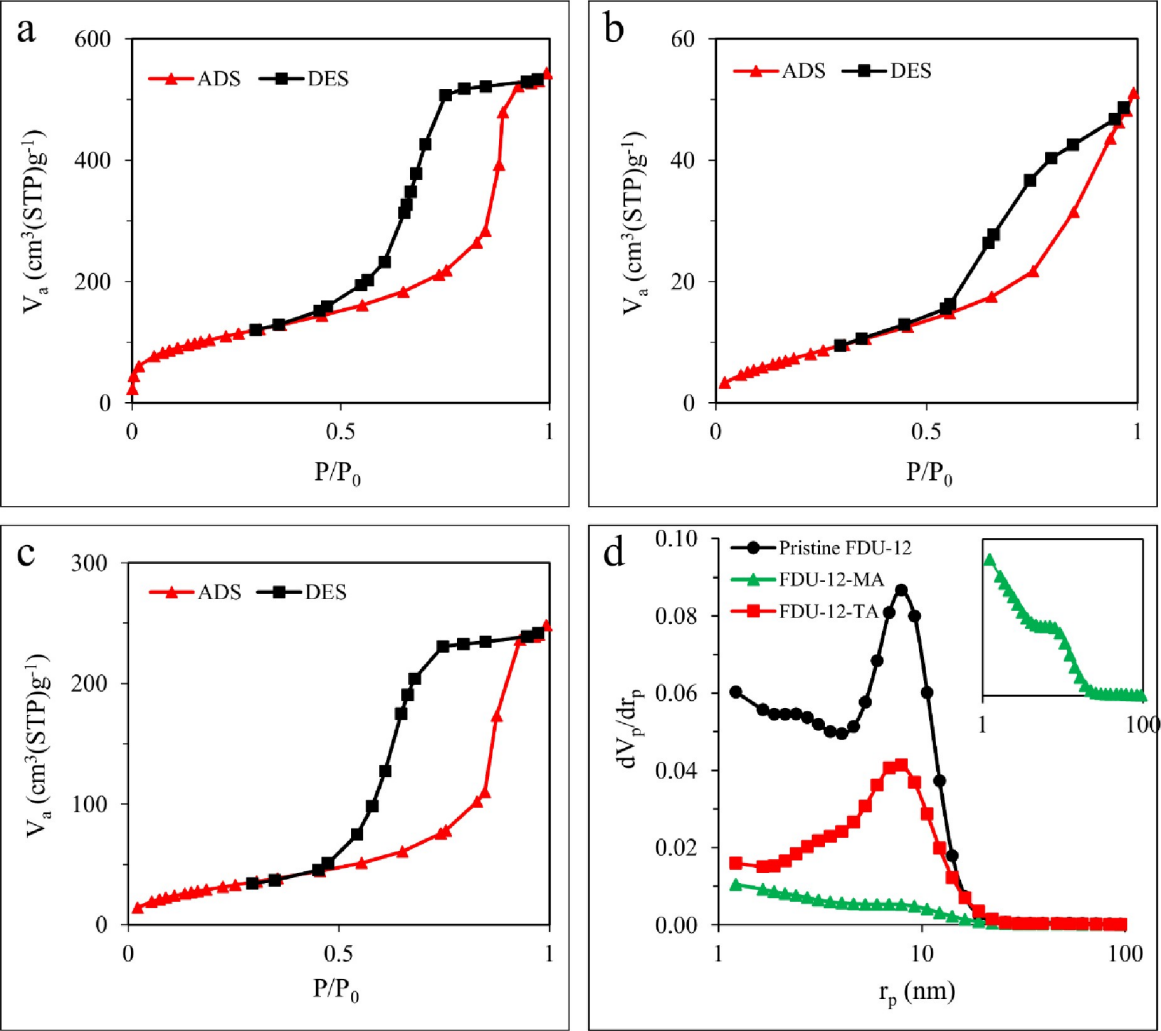
N_2_ adsorption/desorption isotherms of (a) pristine FDU-12, (b) FDU-12-MA, (c) FDU-12-TA, and (d) the BJH pore size distribution curves of the samples.

The nitrogen adsorption/desorption isotherms of the pure FDU-12, FDU-12-MA, and FDU-12-TA materials are shown in Fig 4A–4C. As shown in the figure, according to the IUPAC classification, all three samples exhibited type IV isotherms with H2-type hysteresis loops. The pristine FDU-12, FDU-12-MA, and FDU-12-TA samples showed a BET surface area of 376, 29, and 115 m^2^ g^-1^, respectively. According to the BJH model (Fig 4D), mean pore diameters of 9.2, 1.2, and 6.9 nm were found for pristine FDU-12, FDU-12-MA, and FDU-12-TA samples, respectively. In comparison, the textural parameters of pristine FDU-12 have reduced upon surface modification with silane coupling agents, as it was anticipatable. The obtained textural parameters of the materials are shown in Table 1.

**Table 1:** Textural properties of the synthesized pristine FDU-12, FDU-12-MA, and FDU-12-TA.

Sample	BET			BJH		
	SA^a^	PV^b^	PD^c^	SA	PV	PD
	(m^2^ g^-1^)	(cm^3^ g^-1^)	(nm)	(m^2^ g^-1^)	(cm^3^ g^-1^)	(nm)
Pristine FDU-12	376	0.8387	8.9	326	0.8004	**9.2**
FDU-12-MA	29	0.0787	10.9	38	0.0812	**1.2**
FDU-12-TA	115	0.3831	13.4	135	0.3896	**6.9**

^a^ Surface area

^b^ Total pore volume

^c^ Mean pore diameter

### 3.2. Adsorption studies

#### 3.2.1. The effect of pH on adsorption

**Fig 5:**
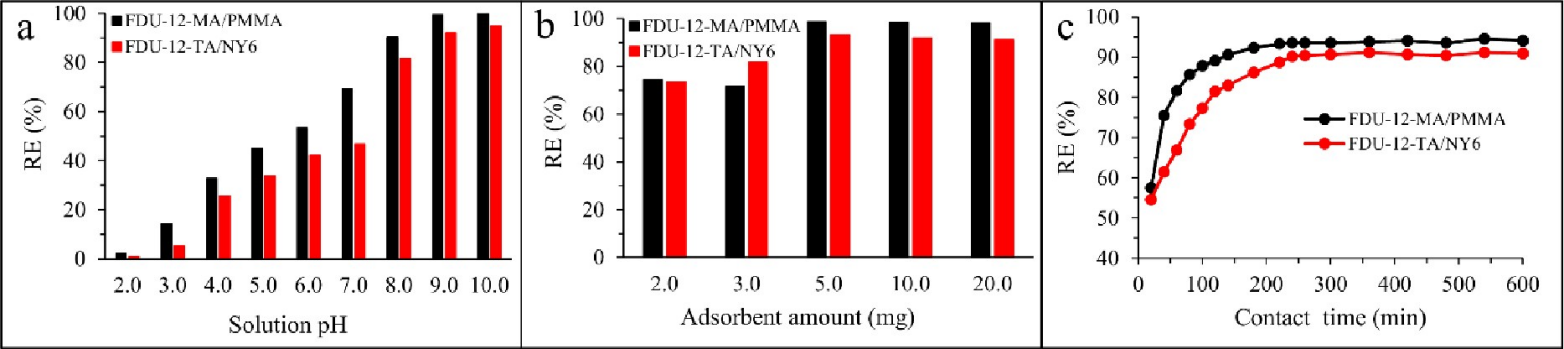
The effect of (a) pH, (b) adsorbent amount, and (c) contact time on adsorption of Pb(II) by the prepared adsorbents.

As an important factor affecting the adsorption process, the effect of pH on the adsorption of Pb(II) ions onto the FDU-12-MA/PMMA and FDU-12-TA/NY6 adsorbents was investigated in the range of 2.0–12.0. The experiments were performed using 10 mL of an aqueous standard solution of Pb(II) at the concentration level of 50 mg L^-1^ with an adsorbent dosage of 10.0 mg. The adsorption was performed at 298 K for 24 h. As can be seen in Fig 5A, for both adsorbents, the removal efficiency was increased with increasing solution pH up to 9.0 and then no significant enhancement occurred. The low removal efficiency in acidic mediums could be related to the competition of H^+^ ions with Pb(II). In other words, the H^+^ ions in the solution occupy the attainable active sites on the adsorbent and compete with Pb(II) ions due to the electrostatic forces. This results in fewer accessible sites for Pb(II) leads to less adsorption of Pb(II) in acidic mediums. Accordingly, pH = 9.0 was selected for further experiments.

#### 3.2.2. The effect of adsorbent dosage

To study the effect of adsorbent dosage on the adsorption of Pb(II) from aqueous solution, various dosages between 2.0 and 50.0 mg were tested. In these experiments, 10 mL of an aqueous standard solution of Pb(II) at the concentration level of 50 mg L^-1^ (pH = 9.0) was used. The adsorption was performed at 298 K for 24 h. The obtained results are shown in Fig 5B. As seen in the figure, the removal efficiency for two adsorbents was enhanced with increasing adsorbent dosage from 2.0 to 5.0 mg. No surprising enhancement in removal efficiency was observed for higher amounts. With enhancing adsorbent dosage, more accessible adsorption sites are available which increases removal efficiency. So, 5.0 mg of each of the adsorbents were used for further experiments.

#### 3.2.3. The effect of contact time

The effect of contact time on the adsorption process was investigated for the two adsorbents. In this step, 10 mL of an aqueous standard solution of Pb(II) at the concentration level of 50 mg L^-1^ (pH = 9.0) with an adsorbent dosage of 5.0 mg was used. The adsorption was performed at 298 K. Fig 5C shows the effect of contact time (20 to 600 min) on Pb(II) removal by FDU-12-MA/PMMA and FDU-12-TA/NY6 adsorbents. Considerable enhancement in removal efficiency was observed when contact time was increased up to 220 and 240 min for FDU-12-MA/PMMA and FDU-12-TA/NY6, respectively. No further adsorption occurred for longer times. The obtained results showed a relatively fast adsorption process. This was mainly due to the high accessible sites on the surface of FDU-12-MA/PMMA and FDU-12-TA/NY6 adsorbents. Based on the results, contact times of 220 and 240 min (for FDU-12-MA/PMMA and FDU-12-TA/NY6, respectively) were selected for further experiments to ensure that equilibrium is reached.

#### 3.2.4. The adsorption kinetic studies

To study the mechanism of adsorption, kinetic studies were conducted. Four kinetic models including pseudo-first-order (PFO), pseudo-second-order (PSO), Elovich, and intraparticle diffusion (IPD) were employed. The applied equations are expressed in the following forms:

$$\log\left(q_{e}-q_{t}\right)=\log q_{e}-\frac{k_{1}}{2.303}t$$ (3)

$$\frac{t}{q_{t}}=\frac{1}{h}+\frac{1}{q_{e}}t$$ (4)

$$h=k_{2}\times q_{e}^{2}$$ (5)

$$q_{t}=\frac{\ln\left(\alpha \beta \right)}{\beta }+\frac{\ln t}{\beta }$$ (6)

$$q_{t}=k_{dif}({t)}^{0.5}+C$$ (7)

**Fig 6:**
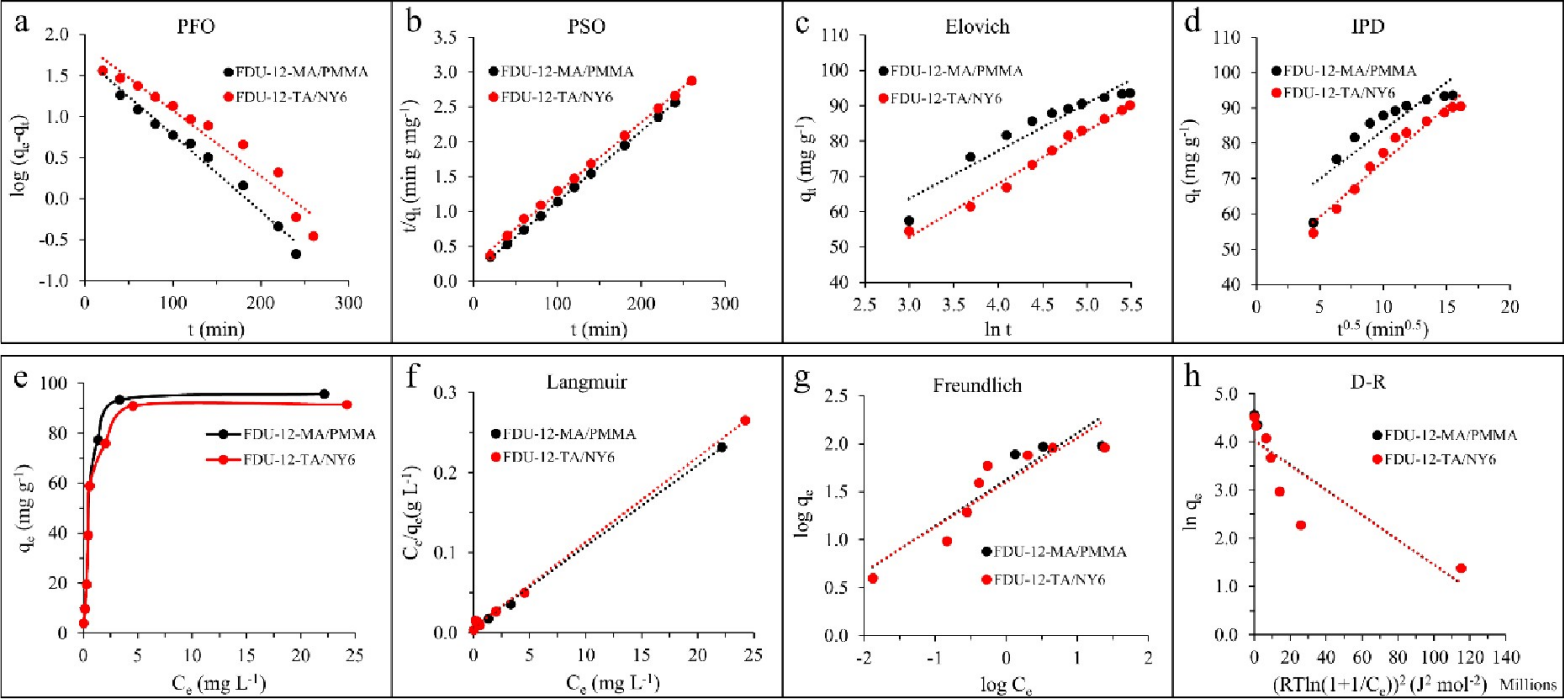
The kinetic adsorption models of (a) pseudo-first-order, (b) pseudo-second-order, (c) Elovich, (d) intra-particle diffusion; (e) the equilibrium isotherm and the isotherm models of (f) Langmuir, (g) Freundlich, and (h) Dubinin–Radushkevich for the adsorption of Pb(II) onto FDU-12-MA/PMMA and FDU-12-TA/NY6.

Where *q*_*e*_ (mg g^-1^), *q*_*t*_ (mg g^-1^), *k*_*1*_ (min^-1^), *h* (mg g^-1^ min^-1^), *k*_*2*_ (g mg^-1^ min^-1^), *α* (mg g^-1^ min^-1^) & *β* (g mg^-1^), *k*_*dif*_ (mg g^-1^ min^-0.5^), and *C* (mg g^-1^) are the adsorption capacity at equilibrium, the adsorption capacity at time t, PFO rate constant, the initial sorption rate in PSO model, PSO rate constant, Elovich constants, IPD rate constant, and a constant, respectively. The results of the fitting are illustrated in Fig 6A–6D and Table 2. As shown in Table 2, in the case of both adsorbents, the PSO kinetic model provided better R^2^ than those obtained by other models, which suggests that the chemical adsorption process can be well described with the PSO model. The presence of hydroxyl, amine, and carbonyl groups on the surface of adsorbents might be involved in the process.

**Table 2:** The parameters obtained by kinetic models for the adsorption of Pb(II) onto FDU-12-MA/PMMA and FDU-12-TA/NY6.

Model	Adsorbent	R^2^	Parameters^a^		
PFO	FDU-12-MA/PMMA	0.9846	*k*_*1*_ = 0.0214	*q*_*e*_ = 51.1	
	FDU-12-TA/NY6	0.9469	*k*_*1*_ = 0.0184	*q*_*e*_ = 74.1	
PSO	FDU-12-MA/PMMA	0.9999	*k*_*2*_ = 0.0008	*q*_*e*_ = 99.0	***h* = 7.6511**
	FDU-12-TA/NY6	0.9983	*k*_*2*_ = 0.0004	*q*_*e*_ = 98.0	***h* = 4.0371**
Elovich	FDU-12-MA/PMMA	0.9149	*α* = 75.5465	Β = 0.0741	
	FDU-12-TA/NY6	0.9884	*α* = 25.0980	β = 0.0665	
IPD	FDU-12-MA/PMMA	0.7883	*k*_*dif*_ = 2.7262	*C* = 56.3580	
	FDU-12-TA/NY6	0.9596	*k*_*dif*_ = 3.1103	*C* = 43.6304	

^a^ The units are as same as mentioned in Section 3.2.4.

#### 3.2.5. The effect of Pb(II) concentration and adsorption isotherm

To study the effect of the initial concentration of Pb(II) on the adsorption behavior, a concentration range between 2.0 and 70.0 mg L^-1^ (pH = 9.0) was investigated. The adsorbent dosage of 5.0 mg of each adsorbent was used for the experiments. The adsorption time was set to 220 and 240 min for FDU-12-MA/PMMA and FDU-12-TA/NY6, respectively. The obtained data are shown in Fig 6E. The equilibrium adsorption studies were investigated by Langmuir, Freundlich, and Dubinin-Radushkevich (D-R) isotherm models. The linear forms of the applied isotherm models are expressed as follows:

$$\frac{C_{e}}{q_{e}}=\frac{1}{q_{max}\times k_{L}}+\frac{C_{e}}{q_{max}}$$ (8)

$$\log q_{e}=\frac{1}{n}\log C_{e}+\log k_{F}$$ (9)

$$\ln q_{e}=\ln q_{max}-B{(RT\;\ln\left(1+\frac{1}{C_{e}})\right)}^{2}$$ (10)

Where *C*_*e*_ (mg L^-1^), *q*_*e*_ (mg g^-1^), *q*_*max*_ (mg g^-1^), *k*_*L*_ (L mg^-1^), *n* & *k*_*F*_ ((mg g^-1^) (L mg^-1^)^1/n^), *B* (mol^2^ kJ^-2^), *R* (j mol^-1^ K^-1^), and *T* (K) are the concentration of Pb(II) at equilibrium, the adsorption capacity at equilibrium, the maximum adsorption capacity of adsorbent, the Langmuir constant, the Freundlich isotherm constants for adsorption capacity and adsorption intensity, Dubinin–Radushkevich isotherm constant, the universal gas constant, and temperature, respectively. The adsorption isotherms and the calculated parameters from the isotherms models are shown in Fig 6F–6H and Table 3, respectively. As can be seen, considering the R^2^ value, the Langmuir model fitted the experimental data (for both adsorbent) better than the other models. The R^2^ values of the Langmuir model were obtained 0.9970 and 0.9978 for FDU-12-MA/PMMA and FDU-12-TA/NY6 adsorbents, respectively. On the other hand, the maximum adsorption capacities of FDU-12-MA/PMMA and FDU-12-TA/NY6 obtained by the Langmuir model were found 99.0 and 94.3 mg g^−1^, respectively. The obtained theoretical maximum adsorption capacities were in good agreement with those obtained in experiments (93.3 and 90.2 mg g^−1^ for FDU-12-MA/PMMA and FDU-12-TA/NY6 adsorbents, respectively). Table 4 shows data from the previous reports for the removal of Pb(II). It can be observed that the FDU-12-MA/PMMA and FDU-12-TA/NY6 adsorbents exhibited promising Pb(II) adsorption capacity when compared to other adsorbents.

**Table 3:** The parameters obtained by isotherm models for the adsorption of Pb(II) onto FDU-12-MA/PMMA and FDU-12-TA/NY6.

Model	Adsorbent	R^2^	Parameters^a^	
Langmuir	FDU-12-MA/PMMA	0.9970	*k*_*L*_ = 1.5781	***q*_*max*_ = 99.0**
	FDU-12-TA/NY6	0.9978	*k*_*L*_ = 1.6308	***q*_*max*_ = 94.3**
Freundlich	FDU-12-MA/PMMA	0.8289	*k*_*F*_ = 42.2474	***n* = 2.0640**
	FDU-12-TA/NY6	0.8378	*k*_*F*_ = 39.4094	***n* = 2.1529**
D-R	FDU-12-MA/PMMA	0.7366	*B* = 2.59 × 10^−7^	***q*_*max*_ = 56.7**
	FDU-12-TA/NY6	0.7429	*B* = 2.57 × 10^−7^	***q*_*max*_ = 55.7**

^a^ The units are as same as mentioned in Section 3.2.5.

**Table 4:** Comparison of the adsorption capacity of FDU-12-MA/PMMA and FDU-12-TA/NY6 toward Pb(II) ions with other adsorbents.

Adsorbent	*q*_*max*_ (mg g^-1^)	pH	Reference
Oxidized multiwalled carbon nanotubes/polypyrrole composite	26.32	6.0	**[51]**
SBA-15-supported Pb(II) imprinted polymer	42.55	6.0	**[52]**
Oil palm bio-waste/multiwalled carbon nanotubes reinforced PVA hydrogel	30.03	7.0	**[53]**
Triamino-functionalized KCC-1/chitosan-oleic acid nanocomposites	168	9.0	**[24]**
Fe_3_O_4_/cyclodextrin polymer nanocomposite	64.5	5.5	**[54]**
FDU-12-MA/PMMA	99.0	9.0	**This work**
FDU-12-TA/NY6	94.3	9.0	**This work**

Based on the Langmuir adsorption isotherm model assumption, there are a fixed number of identical adsorption sites on the saturated monolayer surface of the adsorbent and the energy of adsorption is constant. To predict the adsorption performance, the equilibrium parameter of *R*_*L*_ was calculated for the Langmuir model. It can be defined as follows:

$$R_{L}=\frac{1}{{1+(C}_{e}k_{L})}$$ (11)

**Fig 7:**
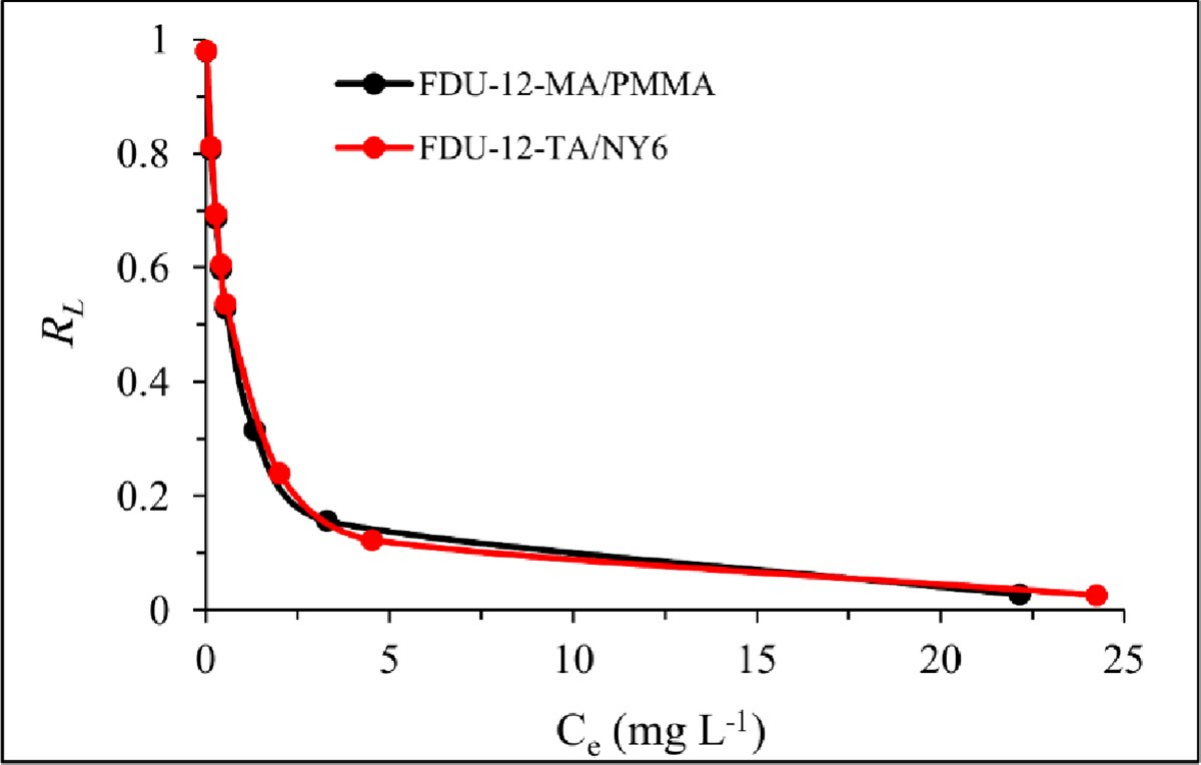
The calculated values of *R*_*L*_.

The *R*_*L*_ value represents the performance of the adsorption process. In this regard, *R*_*L*_ *=* 0, 0 < *R*_*L*_ < 1, *R*_*L*_ = 1, and *R*_*L*_ > 1 suggests irreversible, favorable, linear, and unfavorable adsorption process. The calculated *R*_*L*_ values are shown in Fig 7. All the values were obtained in the range of 0 and 1 which represented favorable adsorption of Pb(II) onto the FDU-12-MA/PMMA and FDU-12-TA/NY6 adsorbents.

## 4. Conclusions

In conclusion, two types of nanocomposites including FDU-12-MA/PMMA and FDU-12-TA/NY6 were fabricated *via* the simple and fast solution polymerization technique. The three-dimensional large mesoporous silica FDU-12 was synthesized and modified with methacrylate and triamine moieties and incorporated into the organic polymers. Due to the relatively large surface area and abundant active sites of the prepared nanofillers, the newly prepared mesoporous silica-based nanocomposites showed good performance toward Pb(II) removal from aqueous solutions. After investigation of the affecting experimental parameters including sample solution pH, adsorbent dosage, contact time, and initial concentration of the metal ion, several kinetic models were studied and the best fit achieved with the pseudo-second-order model for adsorption of Pb(II) using FDU-12-MA/PMMA and FDU-12-TA/NY6. On the other hand, four isotherm models were applied to investigate the equilibrium adsorption studies. Among them, the Langmuir model showed the best fit (R^2^ values of 0.9970 and 0.9978 for FDU-12-MA/PMMA and FDU-12-TA/NY6, respectively). The maximum adsorption capacities of 99.0 and 94.3 mg g^−1^ (by the Langmuir model) were obtained using FDU-12-MA/PMMA and FDU-12-TA/NY6, respectively. In conclusion, the prepared porous nanocomposites showed acceptable characteristics to be considered as effective adsorbents for heavy metals (e.g. Pb(II)) uptake from aqueous media.

## Supporting information

S1 Graphical abstract (TIF)
