# Supplementary figures and images for: Study on novel modified large mesoporous silica FDU-12/polymer matrix nanocomposites for adsorption of Pb(II)
Source: PLoS One. 2021 Jan 22;16(1):e0245583. doi: 10.1371/journal.pone.0245583 (PMC7822333; doi:10.1371/journal.pone.0245583)

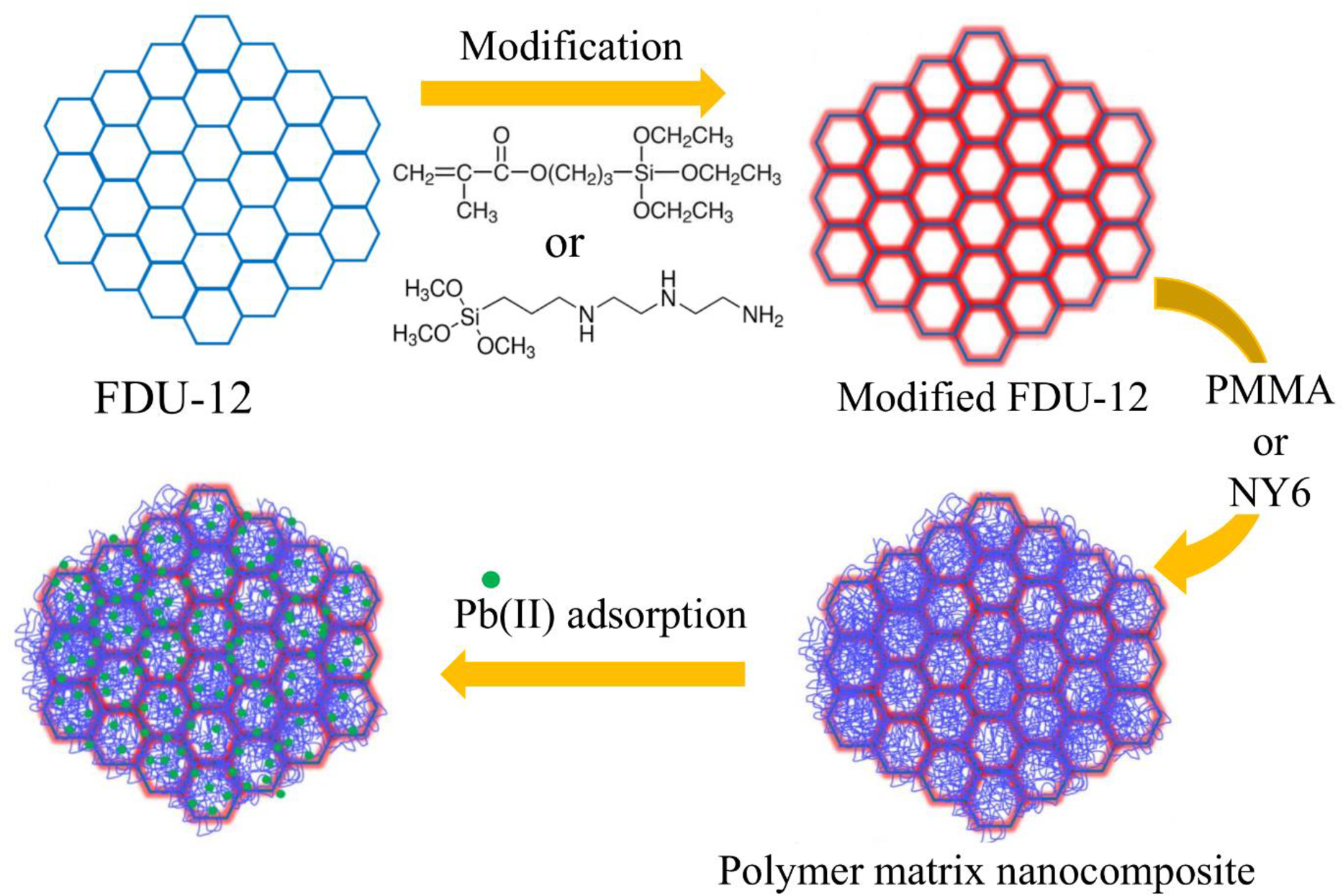

Supplement: S1 Graphical abstract — (TIF) [file pone.0245583.s001.tif]
